# 3D Differential Equation Model for Patients' Choice of Hospital in China

**DOI:** 10.3389/fpubh.2022.760143

**Published:** 2022-04-26

**Authors:** Xiaoxia Zhao, Lihong Jiang, Kaihong Zhao

**Affiliations:** ^1^Faculty of Management and Economics, Kunming University of Science and Technology, Kunming, China; ^2^First People's Hospital of Yunnan Province, Kunming, China; ^3^Department of Applied Mathematics, Kunming University of Science and Technology, Kunming, China

**Keywords:** patients' choice of hospital, number of visits, differential equation model, qualitative analysis, simulation analysis

## Abstract

The number of patients in a hospital is a direct indicator of patients' choice of hospital, which is a complex process affected by many factors. Based on the national medical system and patients' preference for high-grade hospitals in China, this study establishes a three-dimensional differential equation model for calculating the time variation of the number of visits to three grades of hospitals. We performed a qualitative analysis of the system. We carried out a subsequent numerical simulation to analyze the impact on the system when the rate of leapfrog treatment and the maximum capacity of doctors and treatments changed. The results show that the sustainability of China's three levels of hospitals mainly depends on the level of hospital development. The strength of comprehensive health improvement at specific levels is the key to increasing the service efficiency of medical resources.

## Introduction

Hospital selection is the first step for a patient to seek medical treatment, which is a complex process affected by many factors, including the patient's medical behavior, the location of the hospital, the hospital's medical competencies, and the general medical system (1, 2). In the UK, general practitioners complete 90% of outpatient and emergency visits, and the referral rate by general practitioners to specialists is only 5% (3, 4). The number of cases handled by primary medical institutions in the United States, Australia, and Canada exceeds 80% (5–7). Meanwhile, the Chinese medical system is overly dependent on urban large-scale general hospitals for primary medical treatment. Rather than considering the severity of their illness, many Chinese patients choose a hospital based on the comprehensive nature of its medical facilities (e.g., the convenience of location, medical insurance, and other conditions), which is not conducive to the long-term development of the medical system (8–10). The leading cause of this phenomenon is the insufficient supply and uneven distribution of high-quality medical resources in the country and the lack of reasonable procedures available for patient medical treatment (11). To address this issue, the Chinese government began reforming the medical system in 2009. Officials suggested it was necessary to increase the capital input of grassroots-level hospitals and strengthen the training of their medical personnel to balance the allocation of high-quality medical resources and relieve the pressure on large urban hospitals from the high demand for treatment (12, 13).

Considering the scientific literature on patients' choice of hospital, scholars have done questionnaire surveys, and descriptive statistics (14–17), multiple logit and utility maximization nested logit models (2, 18–21), cross-sectional studies (22), demand models (23), dynamic models (24), and game models (25). The process of hospital selection changes with time, and the differential equation model is a suitable method for expressing the temporal nature of this process. It can reveal the internal dynamic relationship of actual events and help us predict future developments, providing a basis for making better decisions. In the fields of economics (26, 27), epidemiology (28), and sustainable science before (29) have applied differential equation models. However, they have had few applications for patients' choice of hospital.

Therefore, based on the preference of Chinese patients for choosing a higher-than-average grade of hospital, this study establishes a differential equation model for hospital selection by patients and analyzes the temporal development of this process assuming that China's medical policy does not change. Because the topological structure of this model is irreversible, it can well describe the influence of patients' high medical preferences on the system. At the same time, the sensitivity of the parameters can be discerned through the numerical simulation of changes in the model parameters to provide a reference for decision makers to allocate medical resources rationally.

The remainder of this paper is organized as follows: Section China's Healthcare System introduces the status quo of China's medical system and patients' medical preferences, section Model Description describes the process of establishing the model, section Equilibrium Points and Stability Analysis provides an analysis of the existence of the equilibrium point under different parameters of the model and an analysis of the conditions of equilibrium stability, section Simulation Analysis details the numerical simulation to prove these analyses, and Section Conclusions summarizes the study and provides some suggestions for medical policy regulations.

## China's Healthcare System

Since the foundation of the People's Republic of China, the Chinese government has been improving the fairness and accessibility of medical resources. However, many issues remain (30, 31). To provide residents with systematic and continuous medical services, the hierarchical design of China's hospitals is highly robust. Hospitals are categorized into three levels based on a comprehensive range of factors (e.g., functions, facility levels, and quality of medical services): first-level hospitals (FLHs) mainly providing daily healthcare services for nearby patients, second-level hospitals (SLHs) providing medical services for patients, and third-level hospitals (TLHs), primarily responsible for treating severe diseases. Since its establishment, China's medical system has made significant progress, the average life expectancy has dramatically improved, and the neonatal mortality rate has significantly decreased.

Nonetheless, China's healthcare system faces extraordinary challenges under the social background of a large aging population, urbanization, and changes in the spectrum of diseases. The allocation of high-quality medical resources in China is imbalanced, with TLHs mainly concentrated in economically developed cities and fewer high-quality medical resources in economically underdeveloped areas. Consequently, one of the most severe problems facing the Chinese medical system is the difficulty and high cost of receiving medical treatment. Not all residents can enjoy high-quality, continuous, and affordable medical services (32–34).

China lacks an effective primary care system (35). Since the late 1970s, the country has not implemented an area-based designated medical treatment policy for residents, so people freely choose hospitals. Thus, patients from different regions and with other diseases can enjoy the same health services, which provides more significant space for the development of medical institutions. However, because the medical resources and the conditions of FLHs are not as good as those of SLHs and TLHs, patients' trust in FLHs is low (10, 36). Many patients use their freedom to seek medical treatment in any establishment to choose SLHs and TLHs that are higher than the level they require, which puts an unreasonable burden on such facilities. It reduces the treatment opportunities for patients with severe illnesses in need of high-quality medical resources and increases the difficulty and cost of treatment. Additionally, the number of visits to SLHs and TLHs has been rising. The volume of diagnoses and treatments in TLHs has declined, resulting in excessive medical resources in SLHs and TLHs and insufficient medical resources in FLHs. Such conduct disrupts the standard order of therapy and is not conducive to the sustainable development of China's medical system.

Another problem is funding. Coupled with low financial investment by the Chinese government, the registration fee and outpatient price of doctors are insufficient to preserve the public service nature of hospitals. Hence, examination, treatment, and medicine have become high, increasing hospitals' revenues. It has led to the Matthew effect between hospitals, that is, the more patients admitted to TLHs and SLHs, the higher the income of TLHs and SLHs, the fewer patients admitted to FLHs, the more serious the shortage of high-quality medical resources, as shown in Table 1 (38, 39).

**Table 1:** The medical resources and services of Chinese hospitals in 2019 (37).

**Classification**	**Number of hospitals**	**Certified doctors**	**Number of beds**	**Number of visits (10,000)**
FLHs	11,264	135,471	651,045	22,965
SLHs	9,687	720,121	2,665,974	134,343
TLHs	2,749	1,030,988	2,777,932	205,701
**Total**	23,700	1,886,580	6,094,951	363,009

Overall, many factors influence the current medical preferences of Chinese residents. Still, if we don't address them, these problems may lead to the collapse of the healthcare system (12, 40). For China's healthcare reform to succeed in the long term, the government must ensure sustainable funding, improve technology in primary hospitals, address the shortage of general practitioners, and make systematic healthcare affordable for citizens.

In 2016, to improve the medical situation in China, the Chinese government issued *Healthy China 2030*, which focuses on improving the fairness of medical care in urban and rural areas to optimize public healthcare systems (36). The policy is an important measure to narrow the gap between China's healthcare system and developed countries and provide residents with the higher health standards of such countries.

## Model Description

The purpose of the Chinese hospital grading design is to enable patients to receive reasonable and continuous medical services according to the severity of their illness. However, the number of patients choosing a higher hospital grade when seeking medical treatment exceeds normal demand, which is not sustainable for the long-term development of China's medical system. If the Chinese government does not intervene, the future of people's choice of hospitals will stunt the progress of development in China's healthcare system.

The number of hospital visits can reflect the choice of hospital. To explore the future state of Chinese patients' choice of hospital, we made assumptions based on the current actual medical conditions in China. We established a differential equation model of the number of visits to hospitals of each level over time. The establishment process of the model is described below.

We assume that the system is closed, and the number of visits is continuous and a differentiable function. We use *x*(*t*), *y*(*t*), and *z*(*t*) for the number of visits to FLHs, SLHs, and TLHs at time *t*, and *x*_0_, *y*_0_, and *z*_0_ to represent the initial value when *t* = 0.

Due to resource limitations, the growth rate of the number of visits also conforms to the natural law of restraining growth, it will gradually decrease with the increase in the number of visits. When the number of visits reaches maximum capacity, its growth rate will be zero and no longer increase. Thus, we use *r*(*x*), *r*(*y*), and *r*(*z*) to represent the growth rate of the number of visits to FLHs, SLHs, and TLHs, respectively. Thus, the following expression is obtained:

(1) $$\begin{array}{r} r\left(x\right)=\{\begin{array}{c} r_{f},x\left(t\right)=0,\\ 0,x\left(t\right)=m_{f}, \end{array} \end{array}$$

(2) $$\begin{array}{r} r\left(y\right)=\{\begin{array}{c} r_{s},y\left(t\right)=0,\\ 0,y\left(t\right)=m_{s}, \end{array} \end{array}$$

(3) $$\begin{array}{r} r\left(z\right)=\{\begin{array}{c} r_{t},z\left(t\right)=0,\\ 0,z\left(t\right)=m_{t}, \end{array} \end{array}$$

where *r*_*f*_, *r*_*s*_, and *r*_*t*_ indicate the inherent increase rate of the number of visits to FLHs, SLHs, and TLHs, and they reflect the number of visits to an ideal state without resource constraints. *m*_*f*_, *m*_*s*_, and *m*_*t*_ represent the maximum capacity of the number of visits of FLHs, SLHs, and TLHs, respectively. Therefore, the above three equations can be expressed as:

(4) $$\begin{array}{r} r\left(x\right)=-\frac{r_{f}}{m_{f}}x\left(t\right)+r_{f}, \end{array}$$

(5) $$\begin{array}{r} r\left(y\right)=-\frac{r_{s}}{m_{s}}y\left(t\right)+r_{s}, \end{array}$$

(6) $$\begin{array}{r} r\left(z\right)=-\frac{r_{t}}{m_{t}}z\left(t\right)+r_{t}. \end{array}$$

Then, the process of the number of visits to the three levels of hospitals changing over time can be expressed as:

(7) $$\begin{array}{r} \{\begin{array}{c} \frac{dx\left(t\right)}{dt}=r\left(x\right)x\left(t\right)=-\frac{r_{f}}{m_{f}}x^{2}\left(t\right)+r_{f}x\left(t\right),\\ \frac{dy\left(t\right)}{dt}=r\left(y\right)y\left(t\right)=-\frac{r_{s}}{m_{s}}y^{2}\left(t\right)+r_{s}y\left(t\right),\\ \frac{dz\left(t\right)}{dt}=r\left(z\right)z\left(t\right)=-\frac{r_{t}}{m_{t}}z^{2}\left(t\right)+r_{t}z\left(t\right). \end{array} \end{array}$$

We know that a substantial number of patients prefer high-level hospitals for medical treatment. We used α to indicate the leapfrog medical treatment rate from FLHs to SLHs, β to indicate the leapfrog medical treatment rate from SLHs to TLHs, and η to represent the leapfrog medical treatment rate from FLHs to TLHs. Accordingly, α*x* represents the number of visits from FLHs to SLHs per unit of time, β*y* represents the number of visits from SLHs to TLHs per unit of time, and η*x* represents the number of visits from FLHs to TLHs per unit of time. Thus, Equation (7) can be rewritten as:

(8) $$\begin{array}{r} \{\begin{array}{c} \frac{dx\left(t\right)}{dt}=-\frac{r_{f}}{m_{f}}x^{2}\left(t\right)+r_{f}x\left(t\right)-\alpha x\left(t\right)-\eta x\left(t\right),\\ \frac{dy\left(t\right)}{dt}=-\frac{r_{s}}{m_{s}}y^{2}\left(t\right)+r_{s}y\left(t\right)+\alpha x\left(t\right)-\beta y\left(t\right),\\ \frac{dz\left(t\right)}{dt}=-\frac{r_{t}}{m_{t}}z^{2}\left(t\right)+r_{t}z\left(t\right)+\eta x\left(t\right)+\beta y\left(t\right). \end{array} \end{array}$$

Additionally, the number of visits will decrease due to deaths in the population and patients abandoning treatment. We define *c*_*f*_, *c*_*s*_, and *c*_*t*_ as the churn rate of the number of visits to FLHs, SLHs, and TLHs, so the loss of their number of visits per unit of time are *c*_*f*_*x*(*t*), *c*_*s*_*y*(*t*), and *c*_*t*_*z*(*t*), respectively. Finally, Equation (8) can be expressed as:

(9) $$\begin{array}{r} \{\begin{array}{c} \frac{dx\left(t\right)}{dt}=-\frac{r_{f}}{m_{f}}x^{2}\left(t\right)+r_{f}x\left(t\right)-\alpha x\left(t\right)-\eta x\left(t\right)-c_{f}x\left(t\right),\\ \frac{dy\left(t\right)}{dt}=-\frac{r_{s}}{m_{s}}y^{2}\left(t\right)+r_{s}y\left(t\right)+\alpha x\left(t\right)-\beta y\left(t\right){-c}_{s}y\left(t\right),\\ \frac{dz\left(t\right)}{dt}=-\frac{r_{t}}{m_{t}}z^{2}\left(t\right)+r_{t}z\left(t\right)+\eta x\left(t\right)+\beta y\left(t\right)-c_{t}z\left(t\right). \end{array} \end{array}$$

where *r*_*f*_, *r*_*s*_, *r*_*t*_ > 0, *m*_*f*_, *m*_*s*_, *m*_*t*_ > 0, *c*_*f*_, *c*_*s*_, *c*_*t*_ > 0, and α, β, η > 0 are certain constants.

This non-linear system (9) describes the changes in Chinese patients' visits to hospitals of this level over time. In the next section, we focus on finding the equilibrium points of the system and analyzing their stability, which is very important for practical applications, because, in reality, the initial data or parameters of each system will inevitably change. According to the analysis results, we can estimate the development status of hospitals at all levels in China.

## Equilibrium Points and Stability Analysis

### Equilibrium Points

The zero solutions of Equation (9) are the equilibrium points. Let $\frac{dx\left(t\right)}{dt}=0$, $\frac{dy\left(t\right)}{dt}=0$, and $\frac{dz\left(t\right)}{dt}=0$. When four non-negative equilibrium points are obtained:

$$\begin{array}{l} (1)\;E_{1}^{*}=\;\left(x_{1}^{*},y_{1}^{*},z_{1}^{*}\right), \end{array}$$

where

(10) $$\begin{array}{r} x_{1}^{*}=0,y_{1}^{*}=0,z_{1}^{*}=0. \end{array}$$

Clearly, this trivial equilibrium always exists, and the practical significance is that there will be no patients choosing a hospital.

$$\begin{array}{l} (2)\;E_{2}^{*}=\;\left(x_{2}^{*},y_{2}^{*},z_{2}^{*}\right), \end{array}$$

where

(11) $$\begin{array}{r} x_{2}^{*}=0,y_{2}^{*}=0,z_{2}^{*}=\frac{m_{t}\left(r_{t}-c_{t}\right)}{r_{t}}. \end{array}$$

We can see that this non-trivial equilibrium exists if and only if *r*_*t*_ > *c*_*t*_, which means that there are $z_{2}^{*}$ patients concentrated in TLHs, and no patients choose FLHs and TLHs.

$$\begin{array}{l} (3)\;E_{3}^{*}=\;\left(x_{3}^{*},y_{3}^{*},z_{3}^{*}\right) \end{array}$$

where

(12) $$\begin{array}{r} x_{3}^{*}=0,y_{3}^{*}=\frac{m_{s}\left(r_{s}-\beta -c_{s}\right)}{r_{s}},z_{3}^{*}=\frac{m_{t}}{{2r}_{t}}(r_{t}-c_{t}\\ +\sqrt{{\left(r_{t}-c_{t}\right)}^{2}+\frac{{4\beta r_{t}m}_{s}\left(r_{s}-\beta -c_{s}\right)}{r_{s}m_{t}}}). \end{array}$$

This non-trivial equilibrium exists if and only if *r*_*s*_ > η+*c*_*s*_, which indicates that there are $y_{3}^{*}$ patients concentrated in SLHs and $z_{3}^{*}$ patients concentrated in TLHs, and no patients concentrated in FLHs.

$$\begin{array}{l} (4)\;E_{4}^{*}=\;\left(x_{4}^{*},y_{4}^{*},z_{4}^{*}\right), \end{array}$$

where

$$\begin{array}{l} x_{4}^{*}=\frac{m_{f}\left(r_{f}-\alpha -\eta -c_{f}\right)}{r_{f}}, \end{array}$$

(13) $$\begin{array}{r} y_{3}^{*}=\frac{m_{s}}{2r_{s}}\left(r_{s}-\beta -c_{s}+\sqrt{{\left(r_{s}-\beta -c_{s}\right)}^{2}+\frac{4\alpha r_{s}m_{f}\left(r_{f}-\alpha -\eta -c_{f}\right)}{r_{f}m_{s}}}\;\right), \end{array}$$

$$\begin{array}{l} z_{4}^{*}=\frac{m_{t}}{{2r}_{t}}(r_{t}-c_{t}\\ +\sqrt{\begin{array}{c} {\left(r_{t}-c_{t}\right)}^{2}+\frac{4r_{t}}{m_{t}}[\frac{\eta m_{f}\left(r_{f}-\alpha -\eta -c_{f}\right)}{r_{f}}+\frac{m_{s}}{2r_{s}}(r_{s}-\beta -c_{s}\\ +\sqrt{{\left(r_{s}-\beta -c_{s}\right)}^{2}+\frac{4\alpha r_{s}m_{f}\left(r_{f}-\alpha -\eta -c_{f}\right)}{r_{f}m_{s}}} \end{array})]}. \end{array}$$

This non-trivial equilibrium exists if and only if *r*_*f*_ > α+η+*c*_*f*_, which indicates that all three levels of hospitals have patients, and their number in each level is $x_{4}^{*}$, $y_{3}^{*}$, and $z_{4}^{*}$. This state of the medical system is better than that of the above three cases.

Let

(*H*_1_) : *r*_*t*_ > *c*_*t*_,

(*H*_2_) : *r*_*s*_ > β + *c*_*s*_,

(*H*_3_) : *r*_*f*_ > α + η + *c*_*f*_.

It is not difficult to draw the following conclusions based on the above discussion.

***Theorem 4.1***. For the equilibrium points of the system (9), the assertions hold as follows:

1. If condition (*H*_1_) holds, then the system has one trivial equilibrium, $E_{1}^{*}$, and one non-trivial equilibrium, $E_{2}^{*}$
2. If condition (*H*_2_) holds, then the system has one trivial equilibrium, $E_{1}^{*}$, and one non-trivial equilibrium, $E_{3}^{*}$
3. If condition (*H*_3_) holds, then the system has one trivial equilibrium, $E_{1}^{*}$, and one non-trivial equilibrium, $E_{4}^{*}$
4. If conditions (*H*_1_) and (*H*_2_) hold, then the system has one trivial equilibrium, $E_{1}^{*}$, and two non-trivial equilibria, $E_{2}^{*}$ and $E_{3}^{*}$
5. If conditions (*H*_1_) and (*H*_3_) hold, then the system has one trivial equilibrium, $E_{1}^{*}$, and two non-trivial equilibria, $E_{2}^{*}$ and $E_{4}^{*}$
6. If conditions (*H*_2_) and (*H*_3_) hold, then the system has one trivial equilibrium, $E_{1}^{*}$, and two non-trivial equilibria, $E_{3}^{*}$ and $E_{4}^{*}$
7. If conditions (*H*_1_), (*H*_2_), and (*H*_3_) hold, then the system has one trivial equilibrium, $E_{1}^{*}$, and three non-trivial equilibria, $E_{2}^{*}$, $E_{3}^{*}$, and $E_{4}^{*}$

### Stability Analysis

This section analyzes the Lyapunov stability of the equilibrium points to predict the future state of China's medical system. We used E^*^ = (x^*^, y^*^, z^*^) to denote the arbitrary equilibrium, and the linearized system of (9) at E^*^ is

(14) $$\begin{array}{r} \frac{du\left(t\right)}{t}=Au\left(t\right), \end{array}$$

where *u*(*t*) = (*x*(*t*), *y*(*t*), *z*(*t*))^*T*^, and


(15) $$\begin{array}{r} A=\left[\begin{array}{ccc} -\frac{2r_{f}}{m_{f}}x+r_{f}-\alpha -\eta -c_{f} & 0 & 0\\ \alpha & -\frac{2r_{s}}{m_{s}}y+r_{s}-\beta {-c}_{s} & 0\\ \eta & \beta & -\frac{2r_{t}}{m_{t}}z+r_{t}-c_{t} \end{array}\right]\\ \left[\begin{array}{ccc} a_{11} & 0 & 0\\ a_{21} & a_{11} & 0\\ a_{31} & a_{32} & a_{33} \end{array}\right], \end{array}$$

The characteristic determinant of the system (9) satisfies the following relation:


(16) $$\begin{array}{r} |\lambda E-A|=0, \end{array}$$

The characteristic equation of System (9) is


(17) $$\begin{array}{l} \lambda^{3}-\left(a_{11}+a_{22}+a_{33}\right)\lambda^{2}+\left(a_{11}a_{22}+a_{22}a_{33}+a_{11}a_{33}\right)\lambda \\ -a_{11}a_{22}a_{33}=0. \end{array}$$

Only if all characteristic roots of the system are negative or the real parts of the characteristic roots are negative is this system locally stable at the equilibrium point. In the following section, we discuss the characteristic roots of each equilibrium point.


$$\begin{array}{l} (1)\;E^{*}=E_{1}^{*} \end{array}$$

The characteristic equation of system (9) is


(18) $$\begin{array}{r} \left(\lambda -r_{f}+\alpha +\eta +c_{f}\right)\left(\lambda {-r}_{s}+\beta {+c}_{s}\right)\left(\lambda -r_{t}+c_{t}\right)=0. \end{array}$$

The eigenvalues of the characteristic Equation (18) are λ_1_ = *r*_*f*_ − α − η − *c*_*f*_, λ_2_ = *r*_*s*_ − β − *c*_*s*_, and λ_3_ = *r*_*t*_ − *c*_*t*_. Thus, $E_{1}^{*}$ is stable only if *r*_*f*_ < α + η + *c*_*f*_, *r*_*s*_ < β + *c*_*s*_, and *r*_*t*_ < *c*_*t*_.


$$\begin{array}{l} (2)\;E^{*}=E_{2}^{*} \end{array}$$

The characteristic equation of system (9) is


(19) $$\begin{array}{r} \left(\lambda -r_{f}+\alpha +\eta +c_{f}\right)\left(\lambda {-r}_{s}+\beta {+c}_{s}\right)\left(\lambda +r_{t}-c_{t}\right)=0. \end{array}$$

The eigenvalues of the characteristic equation (10) are λ_1_ = *r*_*f*_ − α − η − *c*_*f*_, λ_2_ = *r*_*s*_ − β − *c*_*s*_, and λ_3_ = *c*_*t*_ − *r*_*t*_. Thus, $E_{2}^{*}$ is stable if and only if *r*_*f*_ < α + η + *c*_*f*_, *r*_*s*_ < β + *c*_*s*_, and *r*_*t*_ > *c*_*t*_.


$$\begin{array}{l} (3)\;E^{*}=E_{3}^{*} \end{array}$$

The characteristic equation of system (9) is


(20) $$\begin{array}{r} \left(\lambda -r_{f}+\alpha +\eta +c_{f}\right)\left(\lambda {+r}_{s}-\beta {-c}_{s}\right)\\ \left(\lambda +\sqrt{{\left(r_{t}-c_{t}\right)}^{2}+\frac{4\beta r_{t}m_{s}\left(r_{s}-\beta {-c}_{s}\right)}{r_{s}m_{t}}}\right)=0. \end{array}$$

The eigenvalues of the characteristic Equation (2) are λ_1_ = *r*_*f*_ − α − η − *c*_*f*_, λ_2_ = β + *c*_*s*_ − *r*_*s*_, and $\lambda_{3}=-\sqrt{{\left(r_{t}-c_{t}\right)}^{2}+\frac{4\beta r_{t}m_{s}\left(r_{s}-\beta {-c}_{s}\right)}{r_{s}m_{t}}}$. Therefore, $E_{3}^{*}$ is stable if and only if *r*_*f*_ < α + η + *c*_*f*_ and *r*_*s*_ > β + *c*_*s*_.


$$\begin{array}{l} (4)\;E^{*}=E_{4}^{*} \end{array}$$

The characteristic equation of System (9) is


(21) $$\begin{array}{r} \left(\lambda -r_{f}+\alpha +\eta +c_{f}\right)\left(\lambda +\sqrt{{\left(r_{s}-\beta {-c}_{s}\right)}^{2}+\frac{4\alpha r_{s}m_{f}\left(r_{f}-\alpha -\eta -c_{f}\right)}{r_{f}m_{s}}}\right)\\ (\lambda +\sqrt{\begin{array}{c} {\left(r_{t}-c_{t}\right)}^{2}+\frac{4r_{t}}{m_{t}}[\frac{\eta m_{f}\left(r_{f}-\alpha -\eta -c_{f}\right)}{r_{f}}+\frac{m_{s}}{2r_{s}}(r_{s}-\beta -c_{s}\\ +\sqrt{{\left(r_{s}-\beta -c_{s}\right)}^{2}+\frac{4\alpha r_{s}m_{f}\left(r_{f}-\alpha -\eta -c_{f}\right)}{r_{f}m_{s}}})] \end{array}}=0. \end{array}$$

It is clear that $E_{4}^{*}$ is stable if and only if *r*_*f*_ > α + η + *c*_*f*_.

Based on the above discussions of (18) through (21), we can obtain the following significant conditions and conclusions:

***Theorem 4.2***. The following statements are true:

1. If condition (*H*_4_) holds, then $E_{1}^{*}$ is locally asymptotically stable
2. If condition (*H*_5_) holds, then $E_{2}^{*}$ is locally asymptotically stable
3. If condition (*H*_6_) holds, then $E_{3}^{*}$ is locally asymptotically stable
4. If condition (*H*_7_) holds, then $E_{4}^{*}$ is locally asymptotically stable

where

(*H*_4_) : *r*_*f*_ < α + η + *c*_*f*_, *r*_*s*_ < β + *c*_*s*_, *and r*_*t*_ < *c*_*t*_,

(*H*_5_) : *r*_*f*_ < α + η + *c*_*f*_, *r*_*s*_ < β + *c*_*s*_, *and r*_*t*_ > *c*_*t*_,

(*H*_6_) : *r*_*f*_ < α + η + *c*_*f*_ *and r*_*s*_ > β + *c*_*s*_,

(*H*_7_) : *r*_*f*_ > α + η + *c*_*f*_.

**Figure 1:**
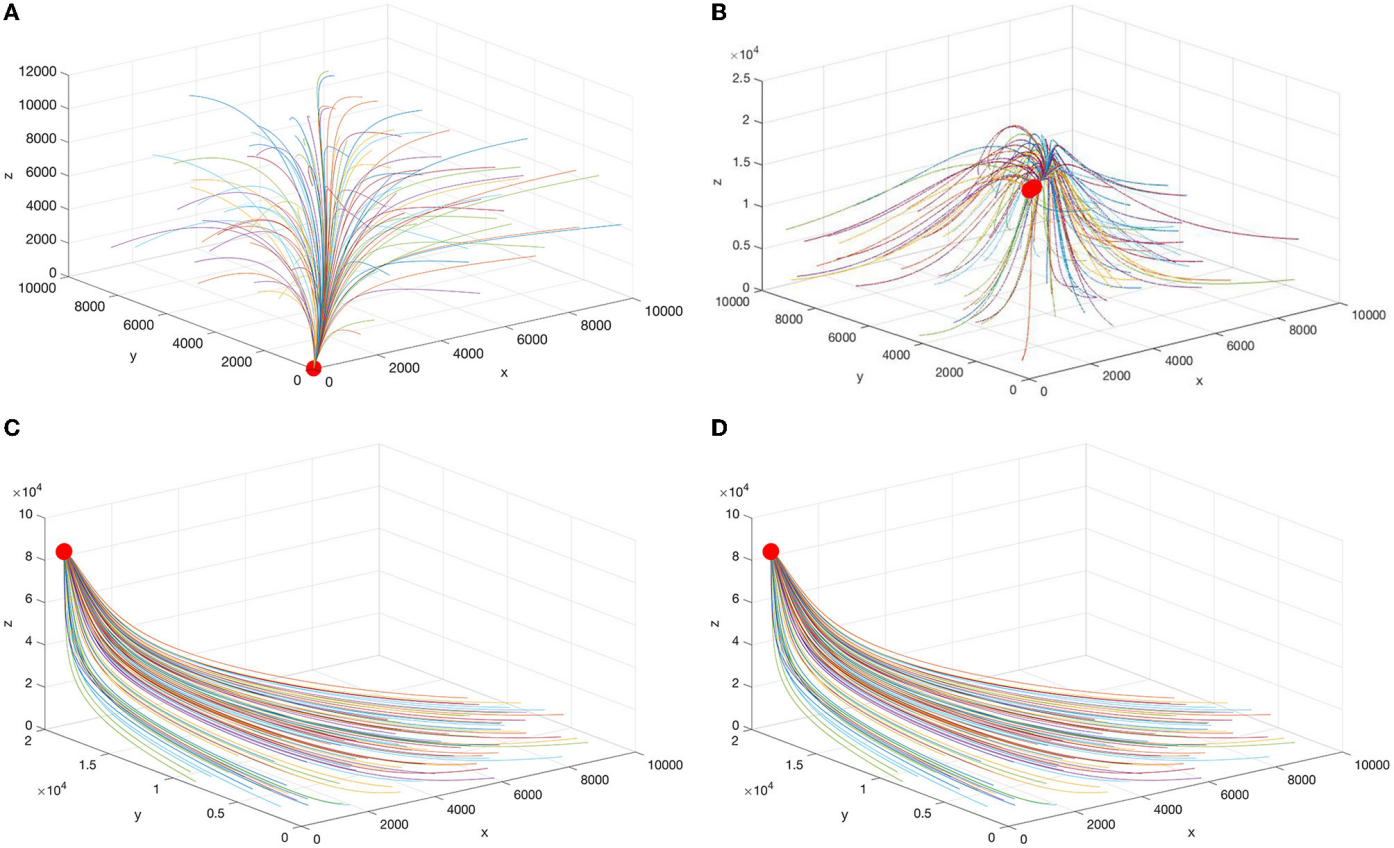
Schematic diagram of the equilibrium points under different parameters. **(A)** $E_{1}^{*}$, **(B)** $E_{2}^{*}$, **(C)** $E_{3}^{*}$, and **(D)** $E_{4}^{*}$.

Figure 1 shows the phase trajectories and equilibrium points for different parameters. In the above discussion, we can see that all three levels of hospitals in China will have patients only if the inherent growth rate of the number of visits to FLHs is greater than the churn rate and the leapfrog medical treatment rate of patients from FLHs to higher hospitals. Therefore, FLHs play a fundamental role in the sustainable development of China's entire medical system.

## Simulation Analysis

### Parameter Fitting

**Figure 2:**
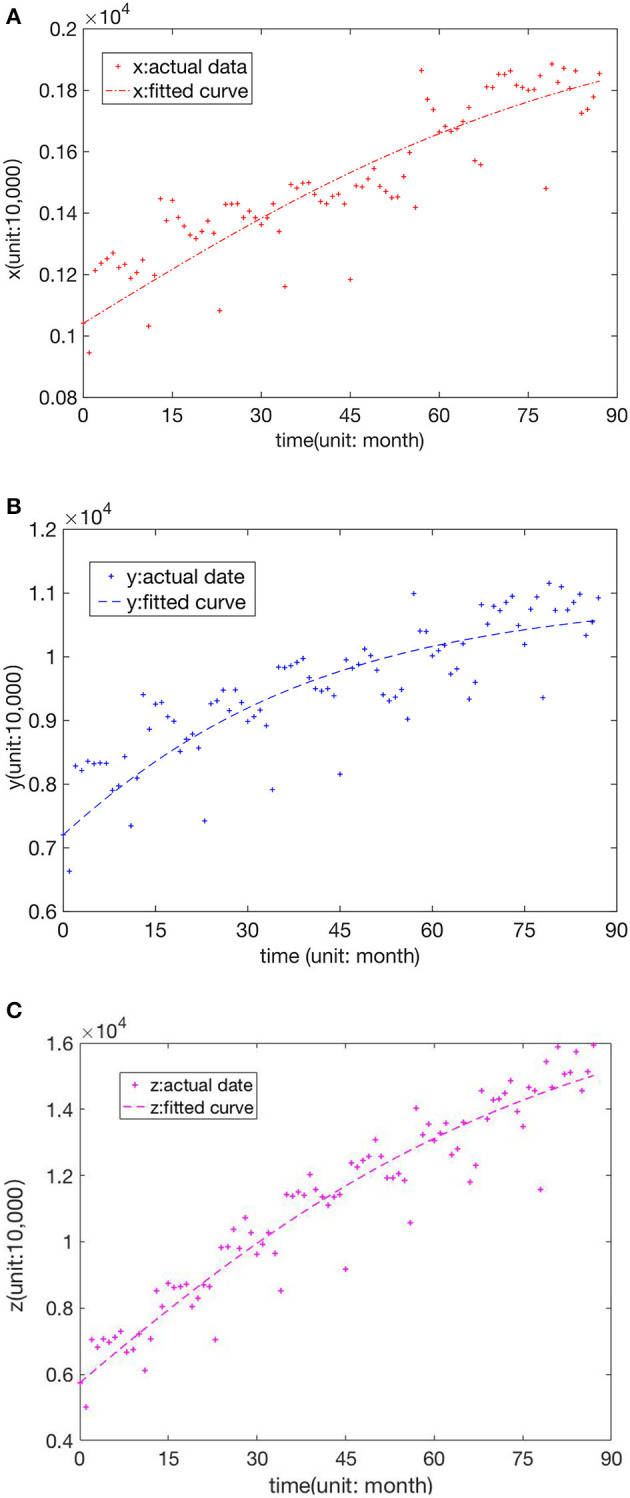
True-date scatter plots and fitting curves of **(A)** FLHs, **(B)** SLHs, and **(C)** TLHs.

This section predicts the future patient distribution among Chinese hospitals based on the current medical treatment situation. We selected the medical data of three levels of hospitals from January to November of each year from 2011 to 2018 from the Chinese Journal of Health Statistics (37), which includes no reports for December of each year. Then, MATLAB software was applied to fit the parameters in Equation (9) by the least square method to obtain α = 0.044, β = 0.037, η = 0.030, *m*_*f*_ = 9000.1, *m*_*s*_ = 29899.2, *m*_*t*_ = 42999.8, *r*_*f*_ = 0.0982, *r*_*s*_ = 0.1096, *r*_*t*_ = 0.0020, *c*_*f*_ = 0.0010, *c*_*s*_ = 0.0406, and *c*_*t*_ = 0.0271. The obtained determination coefficient was *R*^2^ = 0.965, closer to 1, and better fitting, as shown in Figure 2. The actual sampling date has obvious seasonal periodic changes that are not considered in this system.

Note that the data obtained meet the stability condition of the equilibrium point $E_{4}^{*}$, and the number of visits to the three levels of hospitals is calculated to be $x_{4}^{*}=4555.04$, $y_{4}^{*}=13028.5$, and $z_{4}^{*}=18978.6$. There are significant gaps between these and the maximum capacities.

### Parameter Sensitivity Analysis

#### Impact of Leapfrog Medical Treatment Rate

**Figure 3:**
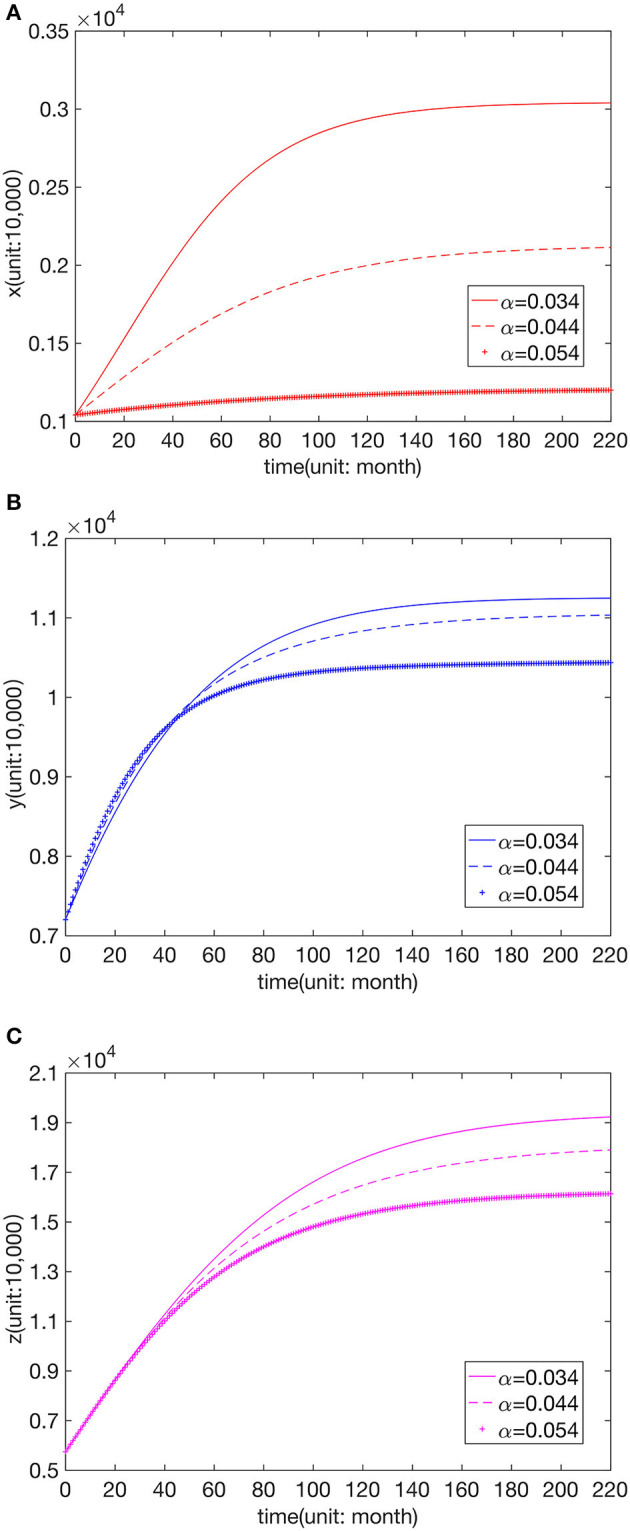
Effect of parameter value α on the number of visits to **(A)** FLHs, **(B)** SLHs, and **(C)** TLHs.

**Figure 4:**
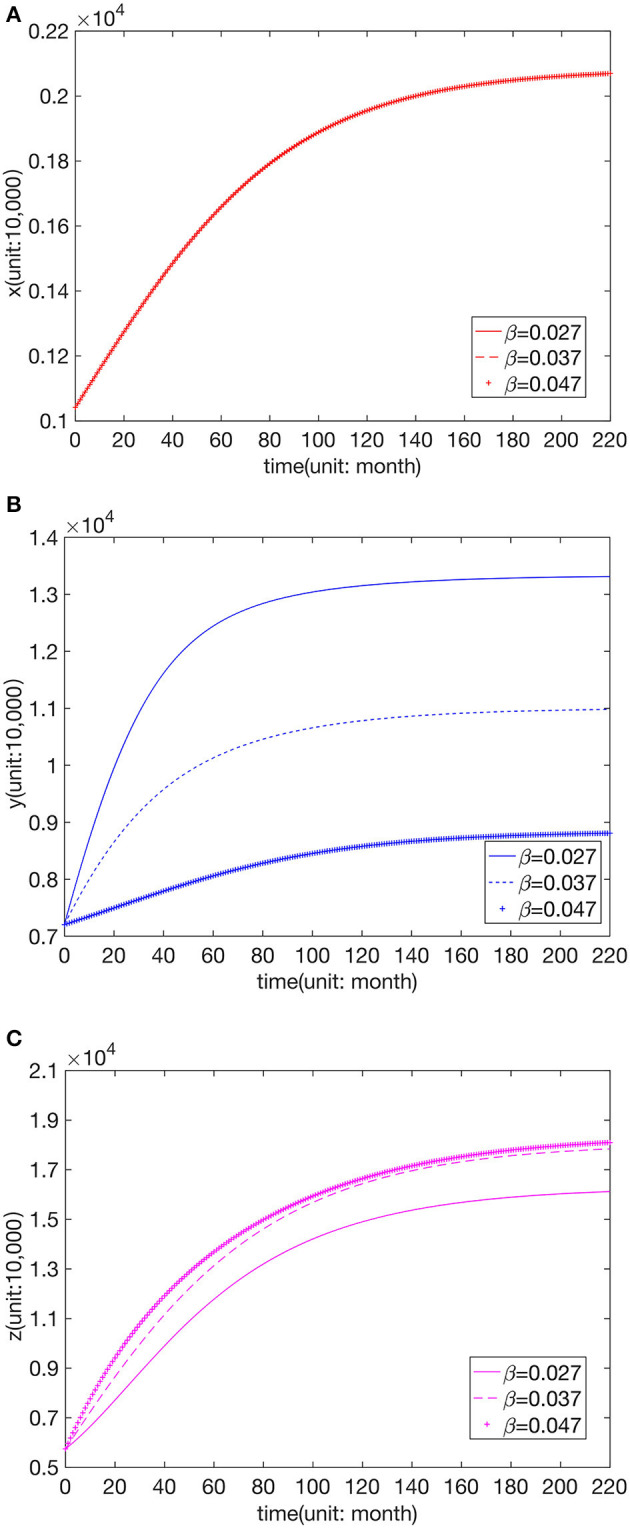
Effect of parameter value β on the number of visits to **(A)** FLHs, **(B)** SLHs, and **(C)** TLHs.

**Figure 5:**
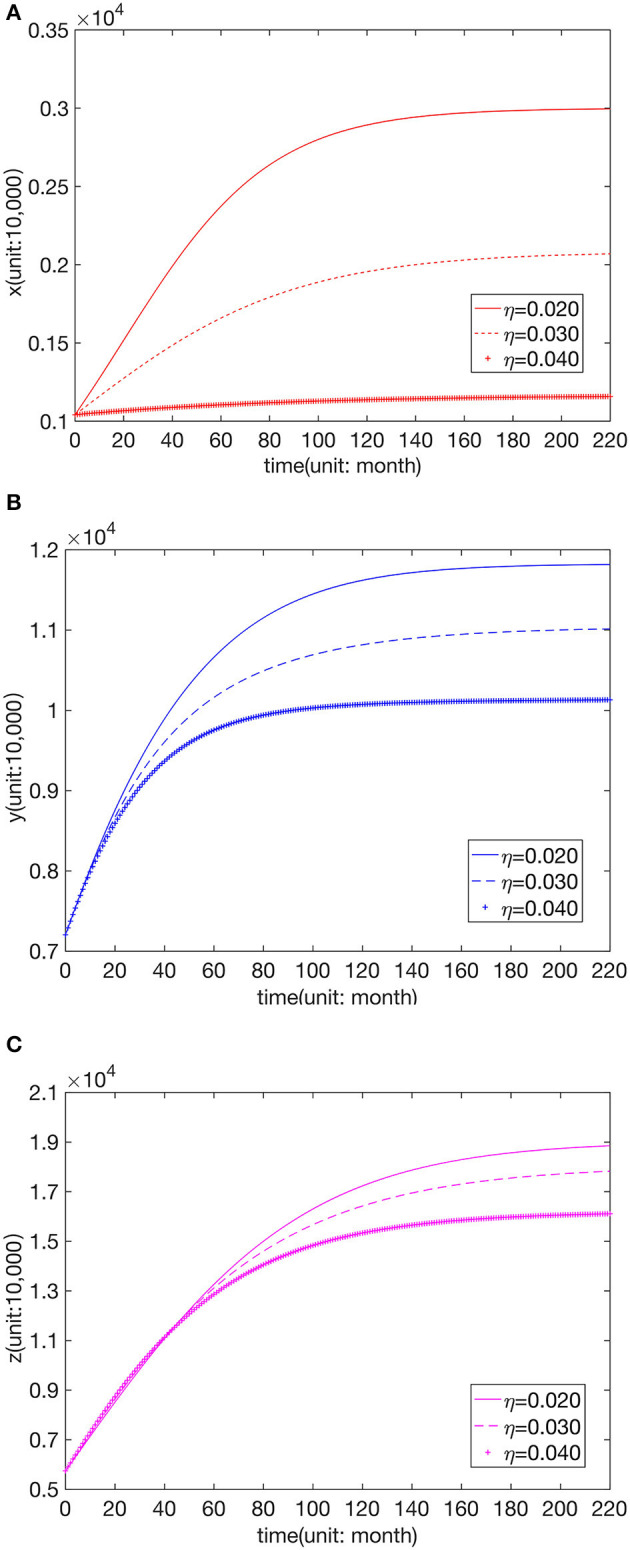
Effect of parameter value η on the number of visits to **(A)** FLHs, **(B)** SLHs, and **(C)** TLHs.

We kept other paraments unchanged to analyze the influence of different leapfrog medical treatment rates in hospitals of all levels. Further, numerical simulation was carried out for the three cases greater than, equal to, and less than the fitting value of the higher hospital-seeking rate, as shown in Figures 3–5.

We can see that the number of visits to hospitals of all levels typically declines with the increase in α, η. The change in β does not affect *x*, and an increase in β leads to a decrease in *y*. When β is greater than or equal to the fitted value, *z* remains unchanged, but when β is less than the fitted value, *z* decreases with the reduction in β.

From the above analysis, it can be seen that the leapfrog medical treatment rate from FLHs to senior hospitals will affect the patient distribution of the entire Chinese medical system, and the increase in the leapfrog consultation rate will reduce the number of visits to all levels of hospitals. When the leapfrog medical treatment rate from SLHs to TLHs decreases, the number of visits to SLHs increases, and that to TLHs reduces. In contrast, the distribution of FLH patients is not affected.

#### Impact of Maximum Visiting Capacity

To analyze the influence of the maximum patient capacity on the number of visits to hospitals at all levels, we kept the other parameters unchanged and conducted a numerical simulation for the three cases with the maximum patient capacities of 0.5, 1, and 1.5 times, respectively.

**Figure 6:**
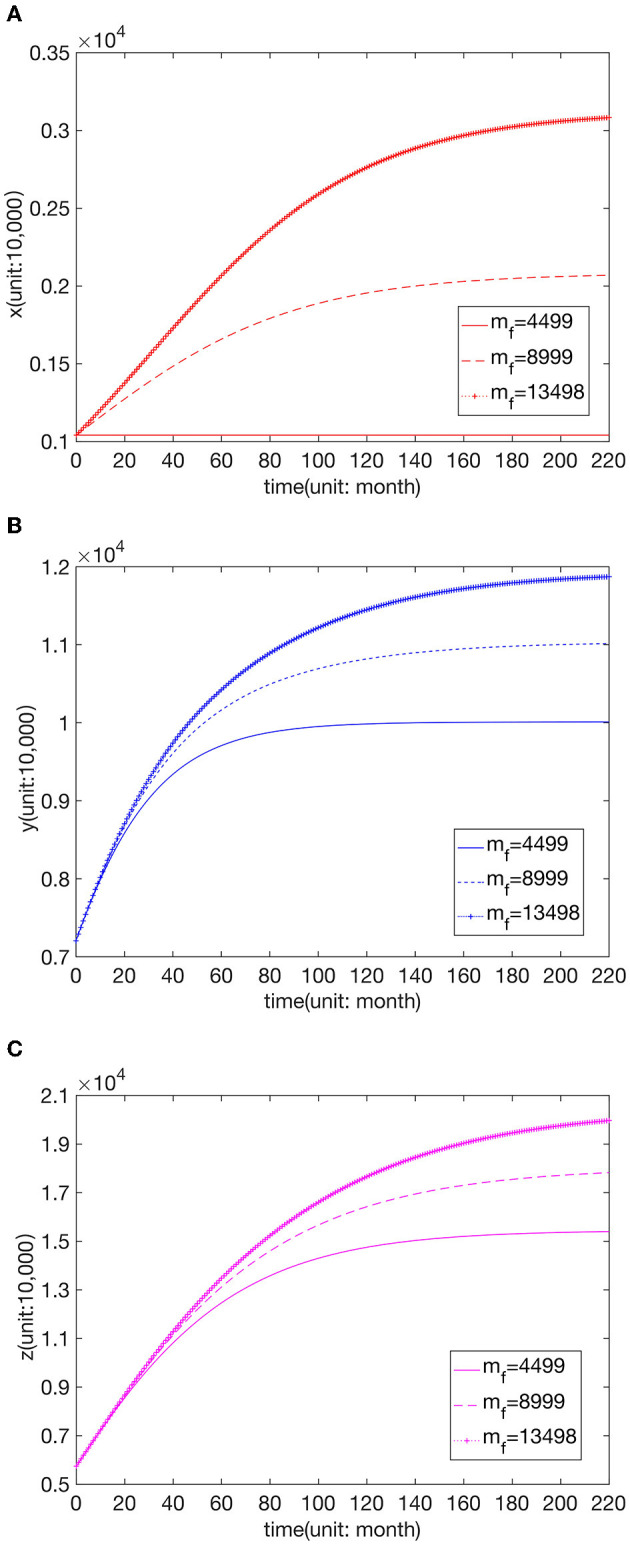
Effect of parameter value *m*_*f*_ on the number of visits to **(A)** FLHs, **(B)** SLHs, and **(C)** TLHs.

**Figure 7:**
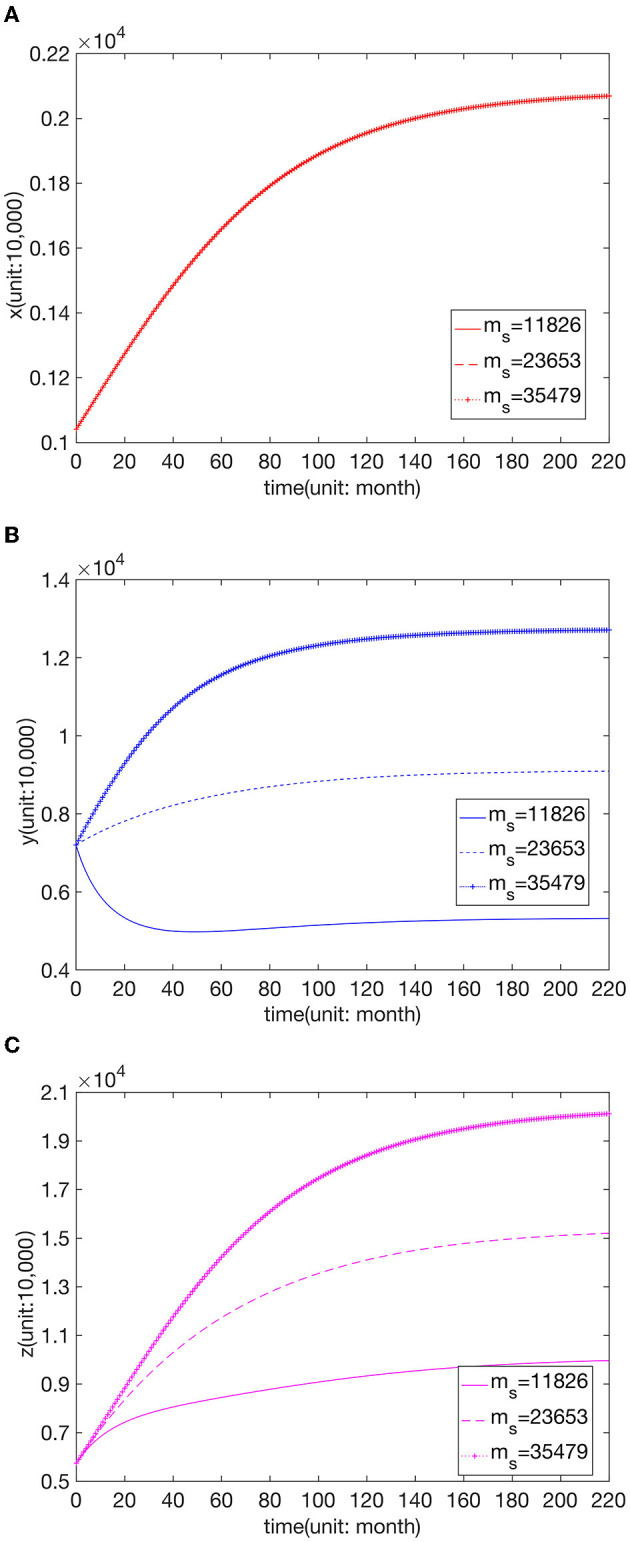
Effect of parameter value *m*_*s*_ on the number of visits to **(A)** FLHs, **(B)** SLHs, and **(C)** TLHs.

**Figure 8:**
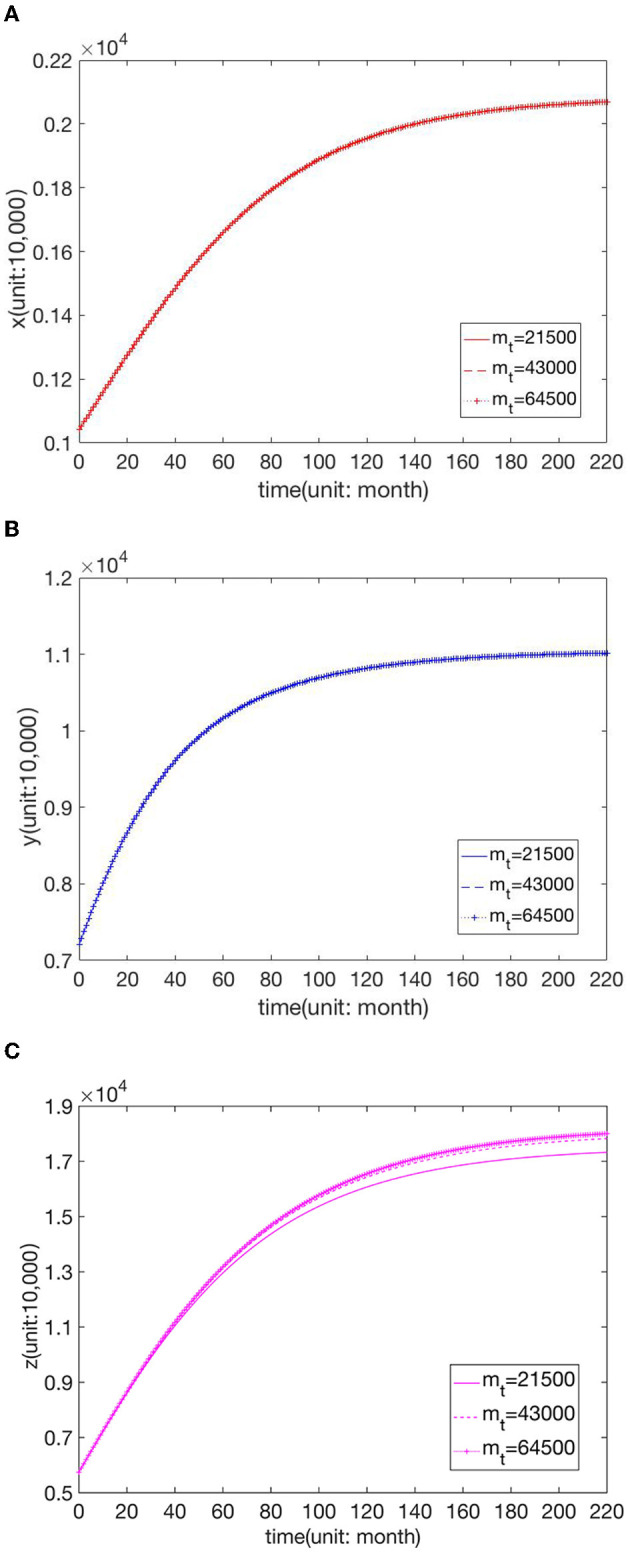
Effect of parameter value *m*_*t*_ on the number of visits to **(A)** FLHs, **(B)** SLHs, and **(C)** TLHs.

We know that the increase in parameters *m*_*f*_, *m*_*s*_, and *m*_*t*_ indicates that the comprehensive strength of the hospitals is improved, and thus the maximum capacity of patients is increased. Figures 6A–C shows the changes in the number of visits to hospitals of the three grades when *m*_*f*_ = 4,499, *m*_*f*_ = 8,999, and *m*_*f*_ = 13,499, respectively. Figures 7A–C presents the changes in the number of visits to hospitals of the three grades when *m*_*s*_ = 11,826, *m*_*s*_ = 23,653, and *m*_*s*_ = 35,479, respectively. Figures 8A–C shows the changes in the number of visits to hospitals of the three grades when *m*_*t*_ = 21,500, *m*_*t*_ = 43,000, and *m*_*t*_ = 64,500, respectively. We can see that the number of visits grows with the rise of *m*_*f*_. And the shift in *m*_*s*_ does not affect *x*, while *y* and *z* increase with the increase of *m*_*f*_. And the change in *m*_*t*_ does not affect *x* and *y*, while *z* increases with the rise of *m*_*t*_, but this effect is not apparent.

## Conclusions

Based on the current data of the Chinese medical system and patients' medical treatment level, this study establishes three differential equation models for the variation of hospital visits over time. It forecasts the development trend of Chinese hospital outpatient visits by analyzing the system's dynamic behavior. Finally, a simulation of the influence of the main parameters on the system was performed. In this manner, we can understand the situation of Chinese patients' choice of hospital and provide a basis for decision-makers to allocate the available medical resources rationally.

In different situations, the system has zero point, boundary equilibrium point, and positive equilibrium point. Among them, the positive equilibrium point means that all three levels of hospitals have patients, which is the basis of the sustainable development of China's medical system. According to the stable condition of the positive equilibrium point, one can see that controlling the rate of patients from FLHs to higher-level hospitals and reducing the loss of patients play a pivotal role in the long-term development of Chinese hospitals. Using previously recorded actual medical data to estimate the model's parameters, we conclude that, in the future development state of China's hospitals at all levels, there will be patients in all three levels of hospitals. Still, the allocation rate of medical resources will be below, and the growth rate of patients in FLHs and SLHs will gradually slow down.

By analyzing the influence of change in the rate of leapfrog medical treatment and the maximum patient capacity of the hospitals on the system, we found that the number of visits to hospitals at all levels in China was negatively correlated with the leapfrog medical treatment from FLHs to higher-level hospitals. The change in the leapfrog medical treatment rate from SLHs to TLHs was negatively correlated with the number of visits to SLHs and positively correlated with the number of visits to SLHs and TLHs, respectively no effect on the number of visits to FLHs. Therefore, from achieving a reasonable distribution of patients, reducing the leapfrog medical treatment in SLHs can enable some patients from TLHs to choose SLHs, thereby relieving the pressure of treatment in TLHs and improving the effective and reasonable utilization of resources.

The increase in the maximum patient capacity of low-level hospitals will also increase the patient capacity of higher-level hospitals. Still, the increase in the maximum patient capacity of higher-level hospitals will not affect the change in the patient capacity of lower-level hospitals. The increase in the maximum patient capacity of SLHs has minimal impact on the overall patient capacity. Therefore, FLHs is the epitome of Chinese hospitals. In other words, with an increase in the comprehensive strength of FLHs, the absolute power of hospitals at all levels will also increase.

Consequently, if the leaders of China's medical system aim to reasonably distribute patients among hospitals at all levels and improve the situation of overcrowding in both large and small hospitals, it is not the best way to control the leapfrog medical treatment rate. Instead, they should improve the comprehensive strength of lower-level hospitals by implementing measures to increase the number of practitioners, enhance their training, and enhance the availability of advanced medical equipment, thereby increasing patients' trust in primary hospitals. Artificial intelligence can be used to improve the service capacity of primary hospitals. For example, in consultation, the system can prompt doctors in primary hospitals to consult patients according to the consultation logic. In the diagnosis process, the system can conduct intelligent analysis and judgment based on the patient's medical record data input by the doctor and assist the doctor in making accurate judgments on the condition. It can also build a new family doctor service model according to the intelligence, understand the health status of residents from time to time, and improve the family doctor compliance rate and residents' satisfaction.

## Data Availability Statement

Publicly available datasets were analyzed in this study. This data can be found at: http://www.nhc.gov.cn/mohwsbwstjxxzx/s2906/new_list.

## Author Contributions

All authors listed have made a substantial, direct, and intellectual contribution to the work and approved it for publication.

## Funding

This study was supported by the National Natural Science Foundation of China nos. 71764035 and 71864021.

## Conflict of Interest

The authors declare that the research was conducted in the absence of any commercial or financial relationships that could be construed as a potential conflict of interest.

## Publisher's Note

All claims expressed in this article are solely those of the authors and do not necessarily represent those of their affiliated organizations, or those of the publisher, the editors and the reviewers. Any product that may be evaluated in this article, or claim that may be made by its manufacturer, is not guaranteed or endorsed by the publisher.
